# Spatial and temporal patterns in the diet of barn owl (*Tyto alba*) in Cyprus

**DOI:** 10.1186/s40709-018-0080-8

**Published:** 2018-05-31

**Authors:** Michaella Moysi, Maria Christou, Vassilis Goutner, Nikos Kassinis, Savvas Iezekiel

**Affiliations:** 1 School of Biology, Aristotle University of Thessaloniki (AUTH), 541 24, Thessaloniki, Greece; 2 Game and Fauna Service, Ministry of the Interior, 1453 Nicosia, Cyprus; 3 Cyprus Association for the Protection of Avifauna, Kalamatas 10 Str., 8047 Pafos, Cyprus

**Keywords:** Barn owl, Cyprus, Feeding ecology, *Tyto alba*

## Abstract

**Background:**

The barn owl, a nocturnal raptor with cosmopolitan distribution, shows a great adaptability to different environments. Regarding prey, the barn owl is a rather selective species, but if changes in the abundance of the selected prey occur, it becomes an opportunistic predator and easily incorporates other prey in its diet, using a wide range of prey species and foraging habitats. Small rodents are usually the prey mostly used. Compared to the populations of north and eastern Europe, barn owl populations in the Mediterranean area have been the least studied. In Cyprus, where barn owl is a common bird species, there are no studies on its diet and feeding ecology. This study was carried out to contribute to the spatial and temporal patterns barn owl diet in Cyprus also providing information on small mammals’ presence and species composition on the island.

**Methods:**

This study was based on 1407 regurgitated pellet analysis that were collected from 26 sites representing six major habitat types on central and southern Cyprus from summer 2013 to summer 2014. The diet of the barn owl was described in terms of seasonal average biomass and numerical percentages of each prey species and compared by Kruskal–Wallis test. Seasonal prey diversity and evenness indices were also calculated. Principal component analysis (PCA) was performed on the prey biomass proportion data assigned to six major habitat types with regard to elevation, vegetation and human uses.

**Results:**

Low prey diversity was found comprised mainly of rodents (overall means 96.2 and 95.7% by number and biomass, respectively). Mice followed by rats were most important prey whereas insectivores, birds and insects were minor components of the owl’s diet. Evenness and diversity values were relatively similar among seasons. PCA differentiated mainly between lowland areas where mice were more abundant prey and mountainous areas where rats dominated in the diet. Insectivores correlated with birds, prey types characterizing several lowland and highland habitats.

**Conclusions:**

The barn owl prey composition in Cyprus suggests an opportunistic foraging behavior, low prey species diversity with variations in the main rodent prey that could be explained by their distribution, seasonal activity and habitat preferences.

## Background

The barn owl (*Tyto alba* (Scopoli, 1769); order Strigiformes) is a nocturnal raptor with cosmopolitan distribution, being common in the temperate and tropical zones of the world [1–3]. It exhibits low resistance to cold [4]. Its presence in arid environments depends on food supply and refuge and enables it to inhabit areas such as Negev desert, Israel [5], Simpson desert, Australia [6] and Atacama desert, Chile [7]. The barn owl shows a great adaptability to different environments explaining its cosmopolitan distribution [7]. As a consequence, use of a wide range of prey species [1, 2] and foraging habitats [8] have been reported. Although foraging opportunism, that is use of a wide range of prey species depending on their availability, has been suggested as a common behaviour exhibited by the species [7, 9–12], it has also been indicated that barn owl is an A selective predator, so its diet does not represent the true abundance of prey in the wild [13–17]. According to Tores et al. [18] and Muñoz-Pedreros et al. [17] the barn owl cannot be defined as a pure opportunist or a pure selective hunter. This strategy of flexible hunting makes the barn owl a very successful predator, explaining its wide cosmopolitan distribution and the ability to colonize new environments, a plastic diet strategy much greater than that of most other species of raptors. In conclusion, barn owl is a rather selective species, but if changes in the abundance of the selected prey occur, it becomes an opportunistic predator and easily incorporates other prey to its diet even prey of low energy value [19], remaining in its territory even when the selected prey types decrease [17]. The barn owl feeds mainly on small mammals (7–24 g), probably without discriminating between large and small prey [20]. In the Mediterranean region, a combination of suitable climatic conditions and long-term human presence has favoured the establishment of this species, supporting relatively high population densities [21]. Compared to the populations in north and eastern Europe, those in the Mediterranean area have been the least studied [8].

Barn owl is a common resident in Cyprus and its population is estimated at 250–750 breeding pairs (these estimates are conservative) [22]. AGROLIFE project [23] in cooperation with Game and Fauna Service explored an alternative to rodenticide rodent control via use of barn owl nesting boxes. In Cyprus, information on the breeding ecology and trends of the barn owl is limited [24], whereas there are no studies on its diet and feeding ecology. Similarly, very few such studies occur in the eastern Mediterranean [10, 25].

The aim of the present study was (a) to contribute to the spatial and temporal patterns barn owl diet in Cyprus, providing information lacking so far; (b) based on prey use of the owl, to provide data on the presence and species composition of small mammals on the island, where relevant information is scarce.

## Methods

### The study area

**Fig. 1:**
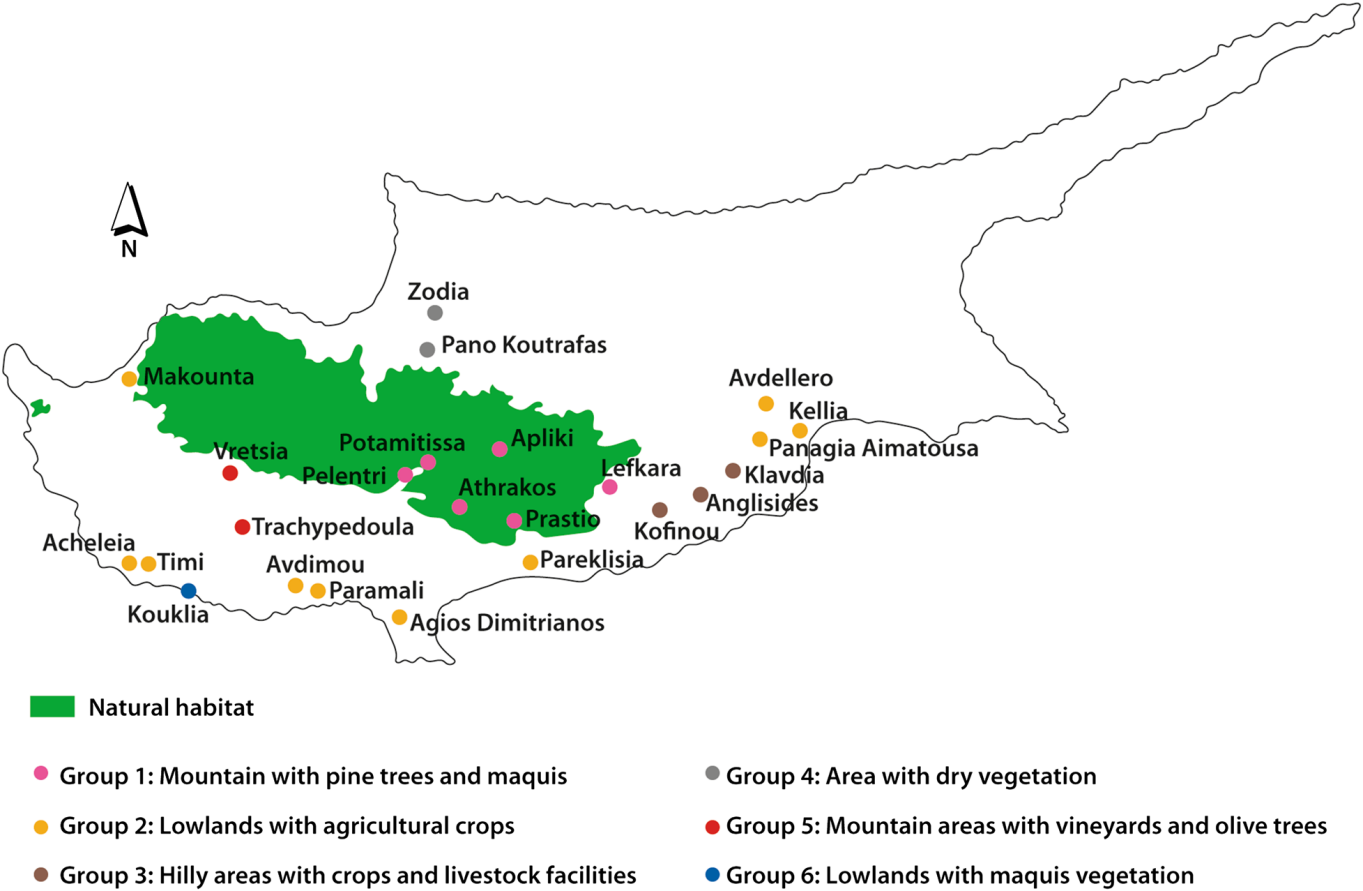
Map of Cyprus indicating the sampling sites of barn owl pellets. Sampling sites have been assigned to groups according to habitat type (1–6)

Cyprus (35°00′N, 33°00′E), is the third largest island in the Mediterranean Sea covering an area of 9250 km^2^ (Fig. 1). The location of the island, with Africa to the south, Turkey and central Europe to the north and the Middle East to the east, is very important for the avifauna of the island. Cyprus includes a diversity of habitats such as sand dunes and rocky coastline, wetlands, streams and lakes, scrubland, agricultural land (e.g. cereal fields, orchards, vineyards), desert-like uncultivated or rocky land, and forests (mostly pine forests) [26].

The locations sampled are presented below. The respective names are indicated in Fig. 1.

### Group 1. Mountain with *Pinus brutia* pine forests and maquis

#### Apliki

A nest was found in a *Quercus alnifolia* zone, with pines and maquis (1000 m asl).

#### Potamitissa

A natural nest was found in a cavity of an oriental plane tree in a clump of trees (alders, plane, pine and olive trees) and scattered low vegetation of thorny shrubs (820 m asl).

#### Athrakos

An artificial nest placed on a pine tree was in use and pellets were collected from a nearby roost. Sparse vegetation with *Cistus* shrubs and some arable land surrounded the sites (676 m asl).

#### Prastio Kellakiou

An artificial nest was in use placed in a hilly area covered with a variety of natural vegetation, such as *Quercus alnifolia*, *Cistus* sp., *Eucalyptus* sp., fruit and pine trees, and cypress trees (490 m asl).

#### Pelentri

An artificial nest was in use situated on a pine tree in an area of scarce pine trees, *Quercus alnifolia*, *Cistus* sp. and fruit trees (800 m asl).

#### Lefkara

An artificial nest was in use situated under a bridge in an area surrounded with pine trees, cypresses trees and reedbeds (430 asl).

### Group 2. Lowlands with agricultural crops

#### Avdimou

An artificial nest on a pine tree in a lowland area was in use. Reedbeds surrounded the area (380 m asl). Another artificial nest placed in the same area on an electric power pole was also in use.

#### Paramali

An artificial nest in use was situated on a cypress tree on cultivated land (20 m asl).

#### Agios Dimitrianos (Episkopi)

Two nests were located on pine trees surrounded by cultivations, scattered pines and fruit trees (23 m asl).

#### Pareklisia

A roost site was found in a rocky area near habitations, sparse vegetation and cultivations with *Pistacia lenticus*.

#### Avdellero

A nest was placed under a bridge within a livestock grazing area (135 m asl).

#### Achelia

Four artificial nests placed on cultivated land were in use (35 asl).

#### Timi

An artificial nest in use was situated in a forest surrounded by cultivations and low vegetation at about sea level.

#### Makounta

A nest was found in a craggy mountainous area. The surrounding habitat was dominated by agricultural and tree cultivations (100 m asl).

### Group 3. Hilly areas with crops and livestock facilities

#### Kofinou

An artificial nest in use was placed near livestock areas. The surrounding vegetation was composed of cultivated trees (mainly almond) and low shrubs (169 m asl).

#### Anglisides

An artificial nest and roost sites in use were under a stone-built bridge. Reedbeds, acacias and olive trees were the most important vegetation around this site (174 m asl).

#### Klavdia

An artificial nest was in use was placed under a bridge. The surrounding habitat was similar to Anglisides (170 m asl).

#### Kellia

An artificial nest in use was placed under a bridge in an area with riparian vegetation of reedbeds and acacias (55 m asl).

#### Panagia Aimatousa

A roost site was located near livestock facilities in an area surrounded by olive trees.

### Group 4. Areas with dry vegetation

One roost site was found near Zodia (85 asl) and three at Pano Koutrafas (241 m asl), in dry craggy areas with bushes, acacias and some fruit tree cultivations.

### Group 5. Mountainous areas with vineyards and olive trees

#### Trachypedoula

A roost was found on a stone-built bridge. The surrounding area included vineyards, olive cultivations and bushland (500 m asl).

#### Vretsia

Two nests were located in deserted buildings in a village. Nearby areas were covered with vineyards and citrus fruit cultivations (500 m asl).

### Group 6. Lowlands with maquis vegetation

#### Kouklia

Roosts were located on a bridge and nearby areas surrounded with maquis and olive and carob trees.

### Pellet collection

Available evidence indicates that pellet analysis is still the most suitable method for studying the diet of owls especially the medium sized ones [9, 27, 28]. Analysing a small sample of pellets can give adequate information about prey composition in the field and it takes less working hours than mammal trapping [29, 30]. Despite the controversy whether pellets represent the true community structure of the prey [15], pellets can give information about prey-species communities and other biogeographic data [1, 31, 32]. Pellets are relatively easy to find and small bones remain well preserved within them [2]. Pellet analysis is a useful tool for the management and protection of owl species and their habitats [1] while the outcome from the analysis can be used to assess ecosystem health [2, 33].

Pellets were collected at the end of every season for 1 year, from summer 2013 to summer 2014. They were collected from natural nests (situated at old buildings, tree cavities and under bridges) and nesting boxes placed and monitored by the Game and Fauna Service. The nests were located at a variety of habitats, including coastal areas, lowlands (0–500 m elevation) and mountain areas (above 500 m elevation) with different types of vegetation (see above for a detailed presentation of the localities).

### Data analysis

The pellets were analyzed using reference books [34–36], but excluding those that contained only hair. Mean weight of each prey taxon was taken from the literature [37, 38]. Mice of the genus *Mus* and rats *Rattus* were not identified at the species level because it was impossible to distinguish them by cranial characters. Although their identification could be possible through DNA analysis of hair found in the pellets, this approach was beyond the scope of the present study. Rats most likely belonged to the species *Rattus rattus* as the presence of *Rattus norvegicus* on Cyprus is dubious [36]. Insects were identified at the level of family due to the poor condition of their remains in the pellets. The diet of the barn owl was described in terms of seasonal average biomass and numerical percentages of each prey species. Average prey weight of each species in each period was estimated by multiplying the numbers of each prey item by its mean weight, adding the weights produced and dividing the sum by the total numbers of prey in each sample. The diet of barn owl was analysed for each field sample in terms of numbers and biomass. Median prey weights were compared among different seasons by Kruskal–Wallis test. These tests were performed using R [39] and Statistica version 7.0 (StatSoft, Tulsa, USA) softwares. The prey diversity was estimated at a class level (mammals, birds, insects) by using the antilog of the Shannon-Weiner index [40, 41], while the evenness index for the mammals was calculated by using the Hill’s ratio [42, 43].

The prey types were assigned to six major habitat types with regard to elevation, vegetation and human uses (Fig. 1). Principal component analysis was performed on the prey biomass proportions data from the six habitat types. The analysis showed that 99.6% of the variation in the dataset was explained by the first three components while the first two components explain 77.9% of the variance in the data. Only the first 2 components were considered in the analysis based on the Kaiser stopping rule, i.e. the number of components with eigenvalues over 1 [44].

## Results

### Prey composition and seasonal variation in barn owl’s diet

In a total of 1407 pellets analyzed during the study, 3312 prey items were identified (mean 2.35 prey items per pellet, ranging from 1 to 8). The diet of the species was made up almost exclusively of small mammals, both in number and biomass (overall means 96.2 and 95.7%, respectively) (Table 1). Of the small mammals, mice (*Mus* spp.) dominated the owl’s diet both by numbers and biomass in most seasons, followed by rats (*Rattus* spp.) with their relative proportions varying seasonally (Table 1).

**Table 1:** Seasonal diet of barn owl in Cyprus in % numbers (N) and % biomass (B), from summer 2013 to summer 2014

Prey type	Summer 2013		Autumn 2013		Winter 2013–2014		Spring 2014		Summer 2014	
	N	B	N	B	N	B	N	B	N	B
Insects	2.7	0.1	1.3	0.1	0.4	0.1	0.4	0.1	–	–
Birds	1.8	3.0	4.2	5.8	3.0	3.8	1.7	2.6	3.5	6.0
Mammals	95.5	96.9	94.5	94.1	96.6	96.1	97.9	97.3	96.5	94.0
Mice (*Mus* spp.)	64.6	52.0	70.4	48.8	72.2	44.2	76.9	57.2	76.9	62.3
Rats (*Rattus* spp.)	9.1	36.4	11.9	41.4	16.2	49.5	9.7	36.3	6.6	27.0
Lesser White-toothed shrew (*Crocidura suaveolens)*	20.8	8.4	10.7	3.7	7.5	2.3	9.6	3.6	11.0	4.4
Etruscan shrew (*Suncus etruscus*)	0.9	0.1	1.5	0.2	0.7	0.1	1.7	0.2	2.0	0.3
Total number of prey	331	–	1063	–	759	–	934	–	225	–
Prey diversity	1.24		1.28		1.18		1.12		1.16	
Evenness	0.69		0.71		0.70		0.58		0.60	
Median prey weight (g)	4.86		3.73		2.45		2.30		1.49	
Interquartile range: 25–75%	22.93		26.74		30.40		24.19		21.9	
Average prey weight (g)	14.93		17.29		19.61		16.1		14.8	

Lesser White-toothed shrew (*Crocidura suaveolens*), though numerically higher than rats in summer and autumn of 2013, were much less important by biomass due to their small size. The numerical distribution of these three major prey types differed significantly through the study period (Kruskal–Wallis χ^2^ = 69.638, df = 13, *p* < 0.0001). Etruscan shrew (*Suncus etruscus*) was of minor importance among the mammalian prey present in proportions being highest in summer 2013 and relatively similar in the other seasons (Table 1). Βirds were found in low proportions in the diet (2.6–6.0% by biomass) whereas insects were unimportant as prey (Table 1). Evenness and diversity values were relatively similar among seasons with spring and summer 2014 evenness values being lowest (Table 1).

### Spatial variation in barn owl’s diet

**Fig. 2:**
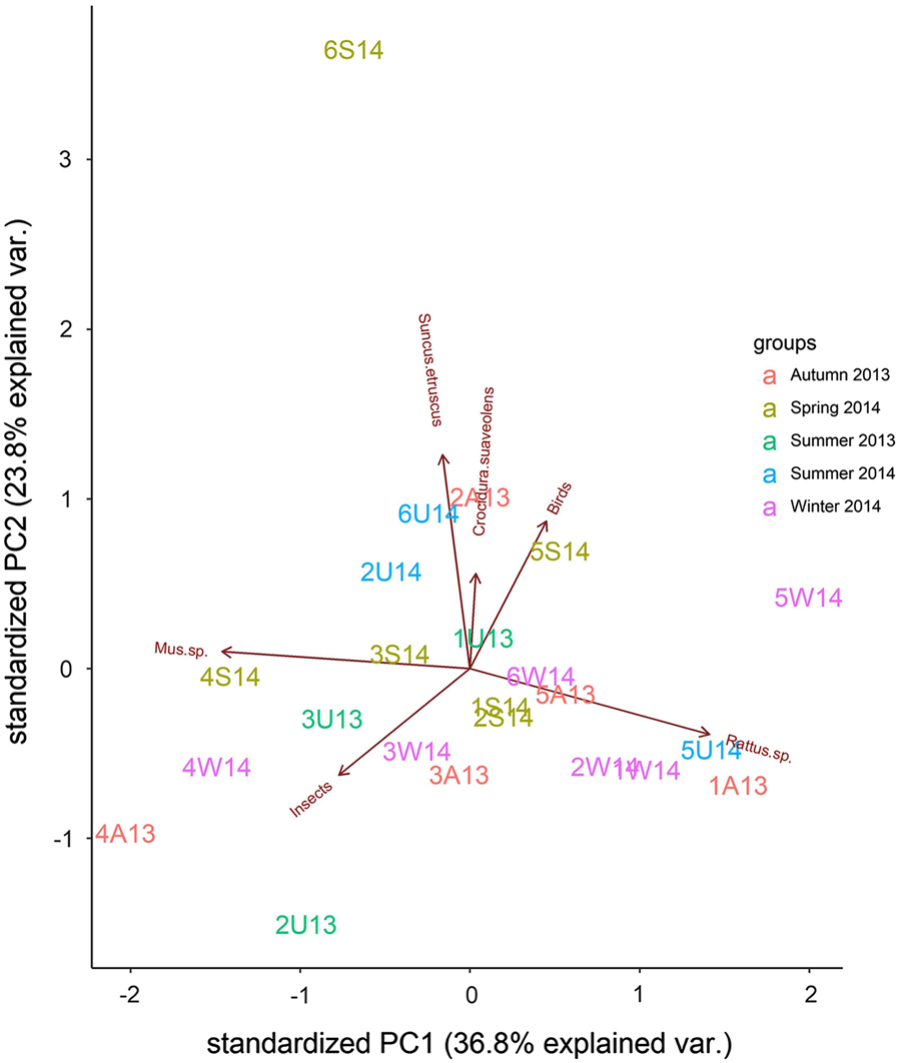
Biplot containing the first two principal components presenting the differentiation of barn owl prey according to habitat. Symbol meaning: 1: Mountain with pine trees and maquis; 2: Lowlands with agricultural crops; 3: Hilly areas with crops and livestock facilities; 4: Areas with dry vegetation; 5: Mountain areas with vineyards and olive trees; 6: Lowlands with maquis vegetation. Different colours denote the 5 different sampling periods during 2013–2014. *A* Autumn, *W* Winter, *S* Spring, *U* Summer (i.e. 2A13 = Samples from lowlands with agricultural crops during autumn of 2013)

PCA biplot (Fig. 2) shows the correlations among prey types and also the habitat and temporal variation in barn owl’s diet. Mice showed a positive correlation with insects and slight to negative correlations to other prey types, particularly rats that were slightly positively correlated only to birds. Etruscan and lesser white-toothed shrews and birds were very highly positively correlated with each other and negatively correlated with insects. Mice and insects were mostly involved in the owl’s diet in hilly areas with crops and livestock facilities, areas with dry vegetation, and lowlands with agricultural crops in both years and across seasons. Nevertheless, insects were not an important prey category (as shown in Table 1). Shrews and birds were more important in lowlands with agricultural crops, lowlands with maquis vegetation and mountain areas with vineyards and olive trees. Rats contributed to the owl’s diet in a variety of habitats, more importantly in mountain areas with pine trees, maquis, vineyards and olive trees and lowlands with agricultural crops.

## Discussion

### Prey diversity and variability

This paper constitutes a novel study of the spatial and temporal feeding habits of the barn owl on Cyprus. Similar studies in the eastern Mediterranean region have only been known for some islands and terrestrial ecosystems of Greece [10, 28, 45, 46] and a limited number of sites sampled from Turkey, NW Syria, SW Lebanon, N Israel, and N Egypt (summarized in [21]). Rodents have been found to be the most common prey in many Mediterranean countries (summarized in [28]). Generally, the composition of barn owl diet in Cyprus reflects that of the eastern Mediterranean area where mammals dominate both by number (95.7% in our study vs. 90%, given by [21]) and by composition [similarly constituting of synanthropic species such as *Mus* spp., *Rattus rattus*, *Crocidura suaveolens*, *Suncus etruscus* and birds (*Passer domesticus*)] [21].

Similarly, to the findings of this study, small mammals comprise the most important prey of the barn owl worldwide, although the prey composition and diversity varies according to the area [ [1, 2, 19, 20, 41, 47–50]; among others]. Among small mammals, two genera of rodents were the major prey of the barn owl in Cyprus followed by two genera of insectivores. Kryštufek and Vohralík [36] reported only five species of Rodentia and two species of Soricidomorpha in Cyprus, being of much lower diversity compared to 15 and 64 species respectively, reported in Turkey. Therefore, the low mammalian prey diversity in barn owl’s pellets in our study reflects the poor mammal diversity of the island, probably explained through the island isolation mechanism [10, 51, 52]. Although rats are considered as the most abundant rodent species on Cyprus [36], in most seasons they were less abundant than mice in the diet of barn owls. Rats presumably compete with house mice [53], therefore various factors contributing to the local availability of the two species might have resulted in their use as prey. Insectivorous mammals have commonly been found as prey for the barn owl [1, 4, 10, 48–50, 54].

It is intriguing that no bats (Chiroptera) were detected in the diet of barn owl in Cyprus. Bats are the second most abundant mammalian group in this region (25% of species, including 17–20 species of bats) [36]. Thus, although potentially available for predation, bats were not taken, and this was probably due to a greater difficulty in capturing them compared to other mammalian prey. Bats have been found in barn owl pellets in the Mediterranean but Obuch and Benda [21] suggested that there was no specialization to bat hunting by the barn owl in this region although some studies would suggest specialization [52, 55, 56]. Birds were a minor constituent in barn owl’s diet. Some studies showed an increase in bird predation when other prey species were rare [2, 10, 57]. In this study, birds were relatively small-sized species (*Turdus* sp.*, Sturnus* sp., *Passer* sp., Fringillidae), roosting mostly in communal perches [58]. An increased bird proportion in the diet during the autumn and summer months may have reflected an increased availability of this prey type, presumably due to the seasonal abundance of juveniles which are probably easier to catch [59] and influx of passage migrants.

### Temporal and spatial prey use

Seasonal trends in the use of the two most important prey types suggest a peak in the use of rats in winter and a decrease in the other seasons, and an inverse situation for *Mus* peaking in spring and summer. A rat prey increase in the winter months might be associated to increased needs for energy intake by the barn owl [60]. Relative prey intake may also reflect the availability of the prey species: the reproductive period of mice may cease during the colder months [12] whereas this of rats is continuous during most of the year [12, 36]. The activity patterns of barn owl and its prey may also play an important role in its participation in the diet as the barn owl is mainly a nocturnal predator and its most important prey is active at night. Thus, a low participation of species such as *Crocidura* could be due to their diurnal activity [38]. Nevertheless, shrews constitute of the most important prey types in the transitory Mediterranean climate zone of southeastern Bulgaria where also mice and rats coexist [48–50]. In this case, most important prey was taken from the predominant dry open cultural land [48, 50]. The relative importance of shrews may also drop due to spikes of other small mammal prey such as voles [49]. Use of rodenticides that cause a decline of the vole and mice population in farms also caused an increase of shrews as the target prey of barn owls [49].

An increase in bird use during autumn and summer may add alternative prey species to compensate for a lower rate of primary prey [7].

In the PCA (Fig. 2), the relationship between habitats and prey types were the most important. The effect of years and seasons was unclear. Mice were important as prey mostly in lowlands with human activities. This seems to be in accordance with the fact that mice on Cyprus have mostly been recorded in disturbed Mediterranean shrubby habitat, dominating only areas under intensive agriculture [36]. The association of shrews (and birds) to lowlands reflects inhabitants of various open habitats where the barn owl prefers to hunt [50]. Nevertheless, these prey types can be taken from higher latitudes where probably are available. The dominance of rats mostly in the samples from mountainous areas seems to reflect the fact that their most important habitat on Cyprus is dense vegetation and plantations while also shrub cover is essential [36].

## Conclusions

The barn owl prey species diversity in Cyprus was low, with variations in the main rodent prey that could be explained by their abundance, distribution, seasonal activity and habitat preferences. The composition of prey indicates an opportunistic foraging behavior that is also reported for many of other eastern Mediterranean populations studied. Each population seems to respond accordingly to its unique features and may either adopt opportunistic feeding habits in response to prey availability or prey selectivity. Further research conducted on the prey population dynamics and biogeography in Cyprus could clarify the seasonal and spatial foraging traits of this nocturnal raptor.

## Abbreviations

PCA: principal components analysis
asl: above sea level
